# Risk Factors for Recurrent Intussusception in Children: A Systematic Review and Meta-Analysis

**DOI:** 10.3389/fped.2019.00145

**Published:** 2019-04-16

**Authors:** Xiaohua Ye, Rong Tang, Shangqin Chen, Zhenlang Lin, Jianghu Zhu

**Affiliations:** ^1^Department of Pediatrics, The Second Affiliated Hospital of Wenzhou Medical University, Wenzhou, China; ^2^The Second School of Medicine, Wenzhou Medical University, Wenzhou, China; ^3^Key Laboratory of Obstetric & Gynecologic and Pediatric Diseases and Birth Defects, Ministry of Education, West China Second University Hospital, Sichuan University, Chengdu, China

**Keywords:** intussusception, recurrence, reduction, risk factor, vomiting

## Abstract

**Background:** Intussusception is a common abdominal emergency in infancy and childhood, and the recurrence rate is reported to be up to 20%. Numerous potential risk factors for recurrence have been reported, although some of them are still controversial.

**Objective:** The present study was conducted to identify the risk factors or predictive symptoms for recurrent intussusception in children who successfully recovered via enema reduction.

**Methods:** The databases of PUBMED, EMBASE, and Cochrane were searched up to August 2018. The primary outcome was the odds ratio involving the following potential risk factors: sex, the presence of blood in stool, fever, abdominal pain, right abdominal mass, pathological lead point, and vomiting.

**Results:** A total of 12,008 participants from 10 studies included in the abovementioned databases were enrolled in this meta-analysis. The correlation strength with each risk factor was as follows: Sex (OR = 0.87 [0.69, 1.09], *P* = 0.22); fever (OR = 1.85 [1.29, 2.65], *P* = 0.0008); blood in stool (OR = 0.93 [0.52, 1.67], *P* = 0.25); abdominal pain (OR = 0.82 [0.49, 1.37], *P* = 0.46); vomiting (OR = 0.55 [0.37, 0.80], *P* = 0.002); pathological lead point (PLP) (OR = 7.71 [1.96,30.29], *P* = 0.003); location of the mass (OR = 0.51 [0.03, 8.28], *P* = 0.64). Besides, children who were relatively older (over 1–2 years of age) were seen to have a higher risk of recurrence.

**Conclusion:** The main conclusion of this meta-analysis was that children with the presence of fever and PLP may have a higher risk of recurrence following enema reduction for intussusception. The prevalence of vomiting was found to be lower in RI (Recurrent Intussusception) patients than in the non-RI patients (control group).

## Introduction

Intussusception is defined as the invagination of a portion of the intestine into itself and is one of the most common causes of abdominal emergencies in infancy and childhood (1), with an incidence of 74 per 100,000 among children <1 year of age (2).

The main methods of treatment for intussusception are enema reduction and operation (3). The surgical method is usually employed as the second line treatment and rarely causes recurrence following successful restitution of the intestine, whereas enema reduction, the primary treatment, has an ~12.7% incidence of recurrence according to a recent meta-analysis (4).

Several studies have reported on numerous potential risk factors, although some of them are still controversial (5–13). Symptoms such as fever and blood in stool and the absence of vomiting have been reportedly associated with recurrent intussusception (RI) in some studies, whereas other studies either did not find any associations or presented a contrary conclusion. Pathological lead point (PLP) was also regarded as a risk factor for RI in some studies (14–18); however, the majority of cases of intussusception were idiopathic, leading to a difficulty in collecting adequate samples from RI patients to verify it with convincing research data.

As the risk factors for RI have not been clearly defined, most hospitals admit patients for 24–48 h of observation (19). However, there is a growing body of literature supporting outpatient management of patients following successful enema reduction (4, 5, 19). Therefore, this meta-analysis was conducted to identify the risk factors or predictive symptoms for RI in children who were successfully treated by enema reduction, and to recognize the children with low risk factors who can be discharged from the hospital.

## Methods and Materials

### Study Design

This meta-analysis was performed according to the Preferred Reporting Items for Systematic Reviews and Meta-Analysis (PRISMA) (20).

### Participants

The RI group included children who had been treated for intussusception successfully by enema reduction and then experienced recurrence. The control group included children who had been treated for intussusception successfully by enema reduction without any recurrence.

### Search Strategy

The PUBMED, EMBASE, and Cochrane databases were searched for articles without restriction of language until January 2019. For extending the range of retrieve, we only set two keywords as the limitation in case of some unexpected missing of any qualified studies. The final strategy is “(intussusception[Title/Abstract]) AND recurren^*^[Title/Abstract]).” It is of note that the reference list of each of the initially included studies was checked to avoid missing any data.

### Study Selection and Data Extraction

The included studies met the following criteria: (1) All participants enrolled were children below 14 years of age. (2) The study groups were clearly defined based on the presence or absence of RI in the participants. (3) Adequate information could be obtained either from full-text screening or was provided by the authors. (4) Participants who received enema reduction did not expose to surgical intervention before the recurrence.

Data such as the first author's name, year of publication, region, potential risk factor assessed in the study, the number of participants, and other useful information were elicited from full-text screening and recorded in Excel tables.

### Statistical Analysis

Literature quality evaluation was conducted by means of the Newcastle-Ottawa Quality Assessment Scale (21). Two reviewers accomplished this process independently, and disagreements were resolved by discussion between them. The presence of publication bias was assessed by visual inspection of the funnel graph.

Odds ratio (OR) was calculated for the outcome involving sex, the presence of blood in stool, fever, abdominal pain, right abdomen mass, PLP, and vomiting. The reason for the choice of OR was the retrospective design of the meta-analysis based on published studies that varied in design, subjects' population, primary outcome measure, and research quality (22).

Forest plot was depicted to present the pooled OR value and heterogeneity was evaluated using *I*^2^ statistic. Due to the potential statistical heterogeneity, a random effect model was chosen to synthesize data instead of a fixed-effect model when assessing the role of sex, blood in stool, vomiting, PLP, and right abdominal mass in developing RI. Heterogeneity was considered significant if *I*^2^ value was over 25%. All reported test results were two-tailed and *P*-value < 0.05 was considered significant. Data analysis was performed with Revman 5.3.

## Results

### Study Selection

**Figure 1:**
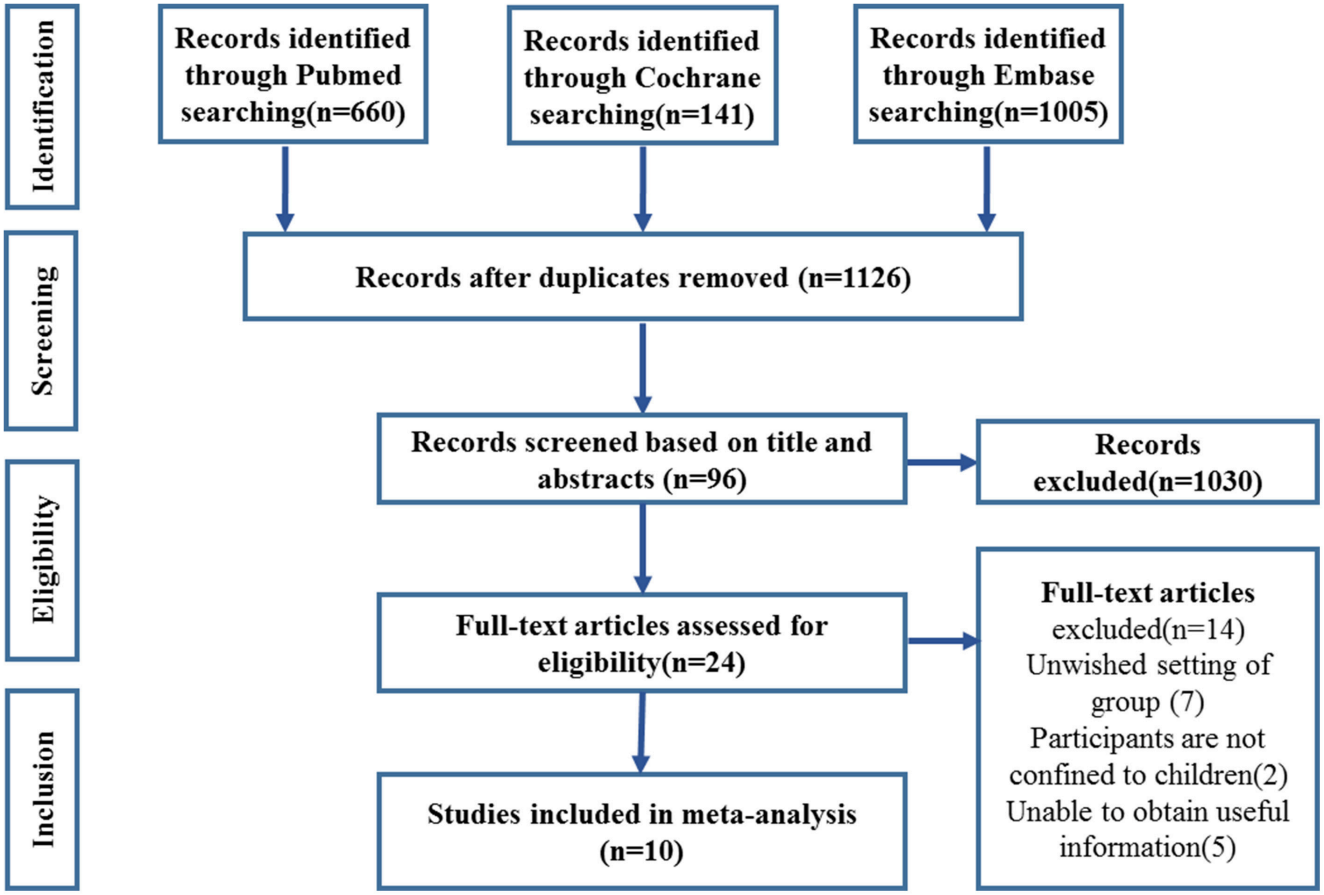
Study selection flow chart.

The flow-chart of this meta-analysis is shown in Figure 1. Initially, studies from three databases (PubMed, Cochrane, and Embase) were retrieved and a total of 1,126 articles were screened based on the title and abstracts after eliminating the duplicates. Further, 24 studies were assessed for eligibility by reviewing full-text articles. Among these articles, seven studies did not compare the outcome of RI patients with a control group whose enema reduction was successful and did not show recurrence (unsuitable setting of group); two did not focus on the pediatric population alone; and five did not provide useful information. Finally, 10 studies were included in this meta-analysis.

### Characteristics of Included Studies

The characteristics of the nine enrolled studies are presented in Table 1. First, except for two American studies, all the others were on the Asian population, which slightly lessened the ethnic confounding factor naturally. Second, the methods for reduction used in these studies were all non-operative; one study used Altrasound-guided saline enema, three studies used barium enema, five studies chose pneumatic enema reduction, and Chen et al. (11) simply mentioned that the method they performed was non-surgical. Third, statistically, all the nine studies chose *P* = 0.05 as the cut-off value for the significance test. Based on that, four studies did not find any significant determinants to predict the presence of RI. Five studies provided the information about how age influences the onset of RI and two reported that the absence of vomiting happens more frequently in RI. Both Guo et al. (6) and Xie et al. (12) investigated the association between blood in stool and RI, but their conclusions were found to be inconsistent, both in the duration of symptoms and location of the mass. However, the presence of an association between PLP and RI was reported in both their studies. Besides, Vo et al. (8) reported that the presence of fever and the sex of the child were prominent factors associated with RI.

**Table 1:** Characteristic table of included studies.

**References**	**Region**	**Method for reduction**	**Positive finding(*P* < 0.05)**
Champoux et al. (10)	America	Barium	None
Chen et al. (11)	China	Non-surgical	None
Guo et al. (6)	China	Air	Higher proportion in RI patients: age > 1 year; duration of symptom < 12 h; without blood stool; without vomiting; right abdomen; with PLP
Kim et al. (7)	Korea	Air	Higher proportion in RI patients: age > 2 years; without vomiting;
Vo et al. (8)	America	Air	Higher proportion in RI patients: fever; female rate
Yang et al. (9)	China	Barium	None
Wang et al. (5)	China	Air	Higher proportion in RI patients: age > 1 year
Esmaeili-Dooki et al. (13)	Iran	Barium	None
Xie et al. (12)	China	Air	Higher proportion in RI patients: age > 2 years; body weight > 12 kg; duration of symptom > 48 h; with blood stool; left abdomen; with PLP
Shen et al. (23)	China	Altrasound-guided saline enema	Higher proportion in RI patients: age > 2 years; the absence of fever

### Risk of Bias

Nine included studies were evaluated by the Newcastle-Ottawa Quality Assessment Scale, which is shown in Table 2. All the included studies had a seven-plus score, which guaranteed the eligibility for meta-analysis.

**Table 2:** Quality evaluation of included studies by Newcastle-Ottawa Scale.

**References**	**Selection of subjects/4**	**Comparability of groups/2**	**Measurement of exposure/3**	**Total score of NOS/9**
Champoux et al. (10)	3	2	2	7
Chen et al. (11)	4	1	3	8
Guo et al. (6)	3	2	3	8
Kim et al. (7)	4	2	3	9
Vo et al. (8)	4	1	3	8
Yang et al. (9)	3	2	3	8
Wang et al. (5)	4	2	3	9
Esmaeili-Dooki et al. (13)	3	2	3	8
Xie et al. (12)	4	2	3	9
Shen et al. (23)	4	2	3	9

Publication bias was assessed via funnel graphs. Based on the symmetry of the funnel graphs, it was less likely that this meta-analysis had publication bias in the models for abdominal pain, blood in stool, and sex, although it showed a high susceptibility for publication bias in the other models.

### Sex

**Figure 2:**
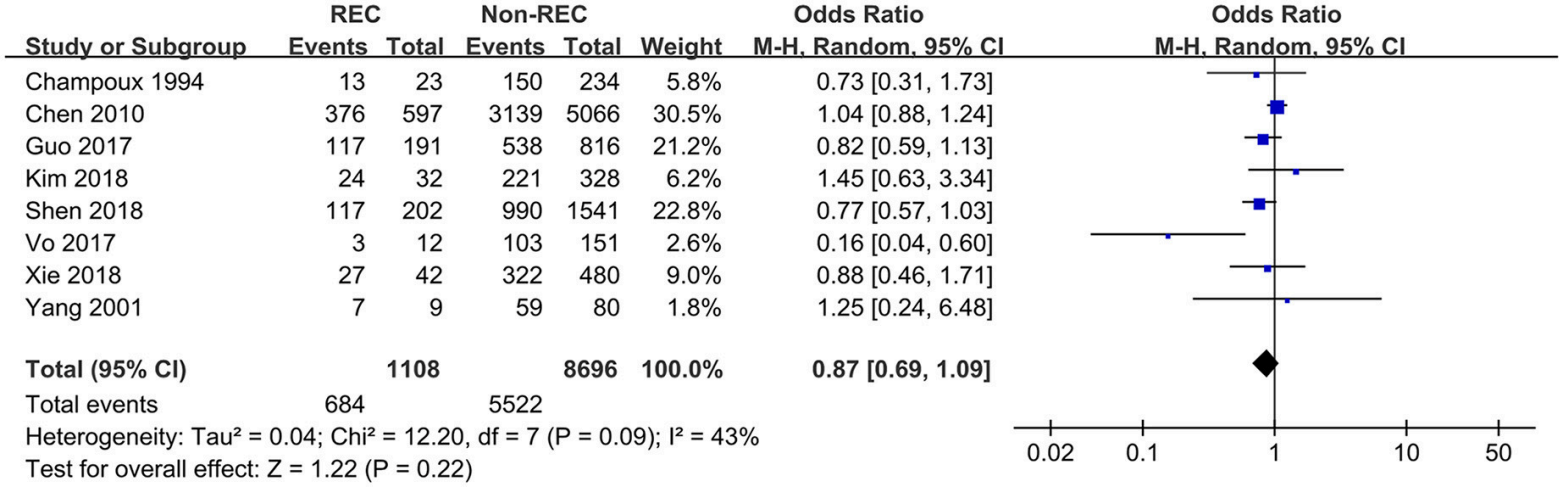
Forest plot showing pooled analyses of sex and RI.

Data pertaining to the female/male ratio were extracted from seven studies and presented as the incidence rate in males. Only Vo et al. (8) reported a higher incidence of RI in female patients, while other studies did not show any differences. This meta-analysis showed that there was no association between sex and the presence of RI, statistically (OR = 0.87 [0.69, 1.09], *P* = 0.22) (Figure 2).

### Fever

**Figure 3:**
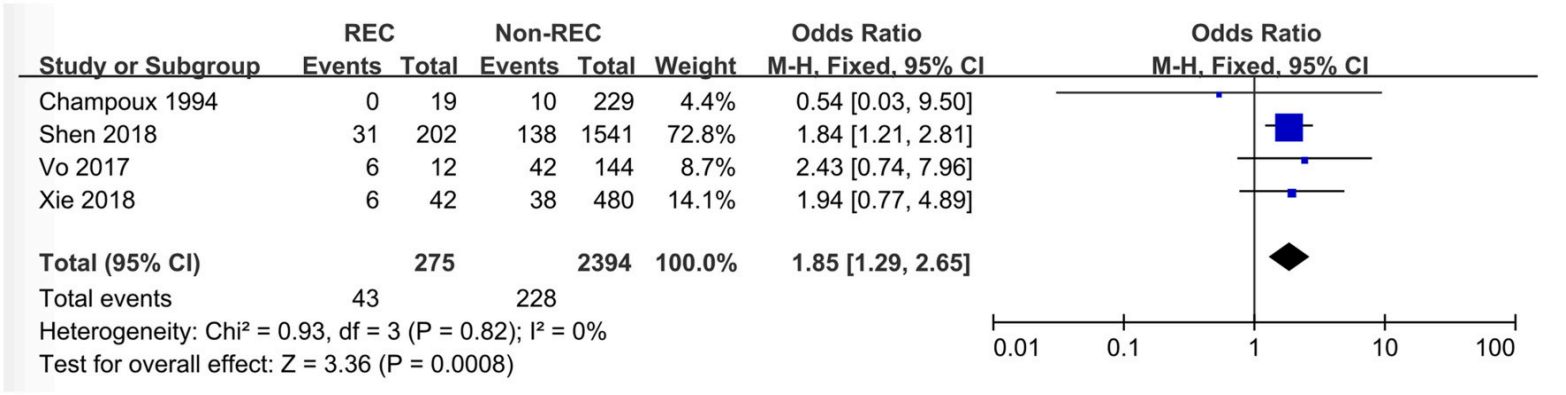
Forest plot showing pooled analyses of fever and RI.

Information about the presence of fever in the participants was extracted from three studies. Both Champoux et al. (10) and Vo et al. (8) chose 38°C (100.4°F) as the cut-off value for fever, which facilitated the integration of data without heterogeneity. Although Xie et al. (12) considered 37.8°C as the cut-off value for fever, the present study still pooled their data because it would not affect the outcome. Kim et al. (7) processed their data by comparing the mean value of body temperature, which hindered the statistic combination with the dichotomous variable in the other studies. Finally, this meta-analysis showed the presence of fever was a valuable risk factor to predict the RI (OR = 1.85 [1.29, 2.65], *P* = 0.0008) (Figure 3).

### Blood in Stool

**Figure 4:**
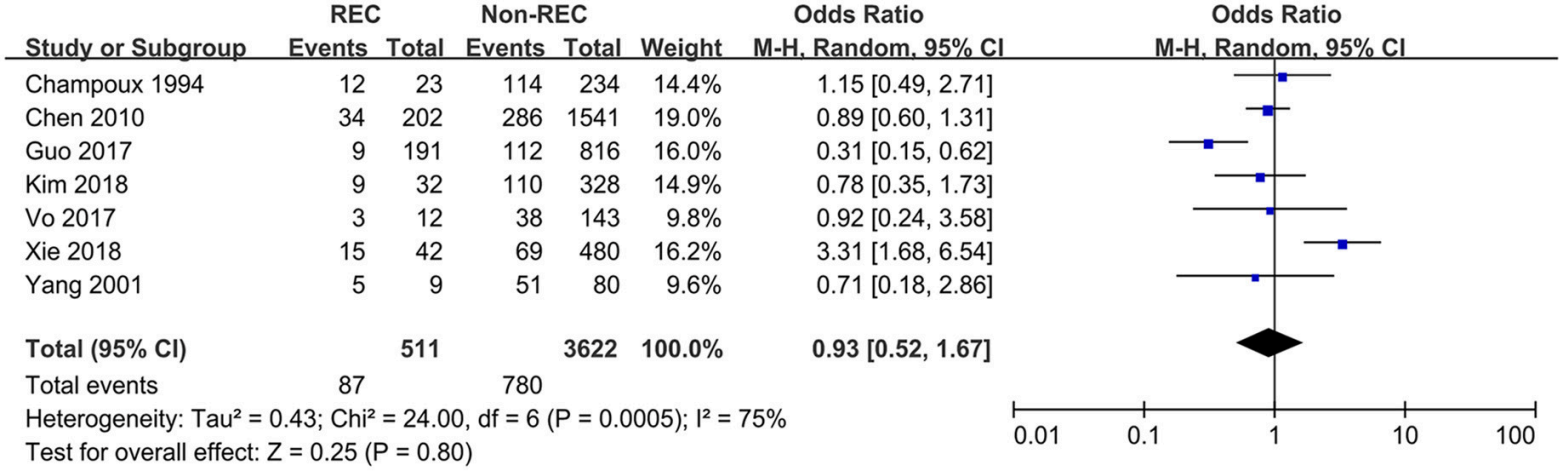
Forest plot showing pooled analyses of blood stool and RI.

Data pertaining to blood in stool were collected from six studies. Guo et al. (6) found the prevalence of blood in stool to be higher in the control group, while Xie et al. (12) reported a contrary conclusion. The other four studies did not find any relationship. This meta-analysis showed that blood in stool was not a predictive symptom of RI (OR = 0.93 [0.52, 1.67], *P* = 0.25) (Figure 4).

### Abdominal Pain

**Figure 5:**
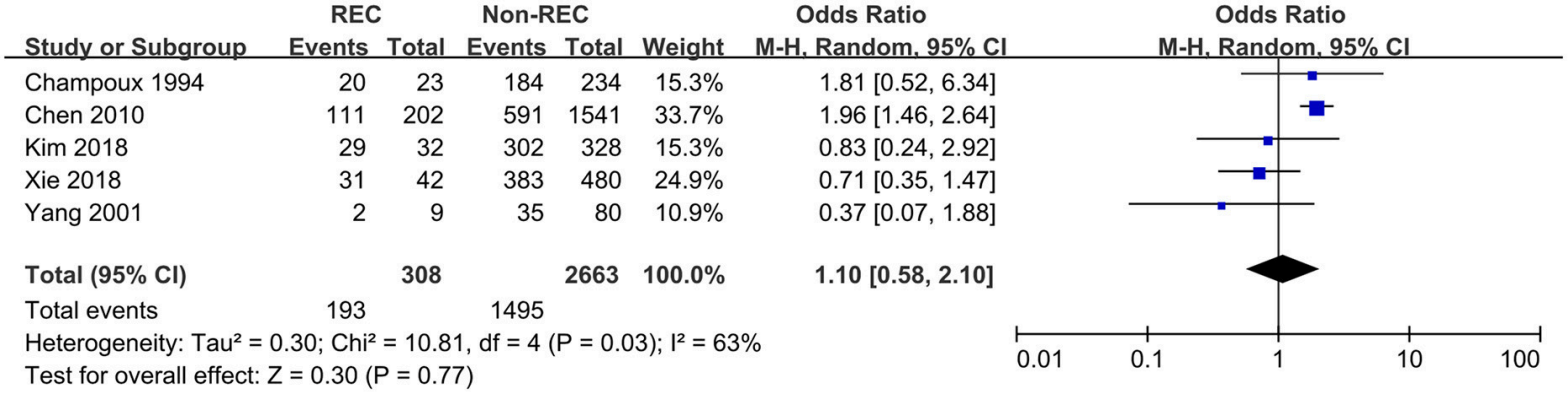
Forest plot showing pooled analyses of abdominal pain and RI.

Detailed data about the abdominal pain were extracted from four studies, and all of them did not present a positive finding. Accordingly, this meta-analysis showed that abdominal pain was not a key symptom in distinguishing RI patients from Non-RI patients (OR = 0.82 [0.49, 1.37], *P* = 0.46) (Figure 5).

### Vomiting

**Figure 6:**
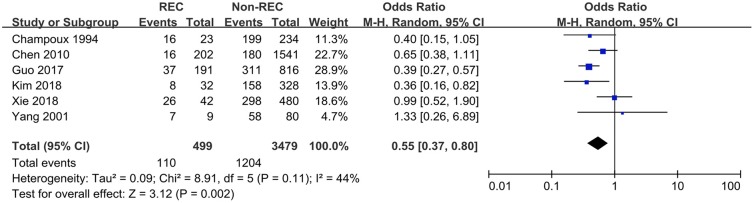
Forest plot showing pooled analyses of vomiting and RI.

Data related to the presence of vomiting in the participants were extracted from five studies. Guo et al. (6) and Kim et al. (7) reported a lower prevalence of vomiting in RI patients, while the other studies did not find any statistic association. The synthesized endpoint of this meta-analysis showed that the prevalence of vomiting was lower in RI patients compared with that in the control group (OR = 0.55 [0.37, 0.80], *P* = 0.002) (Figure 6).

### PLP

**Figure 7:**
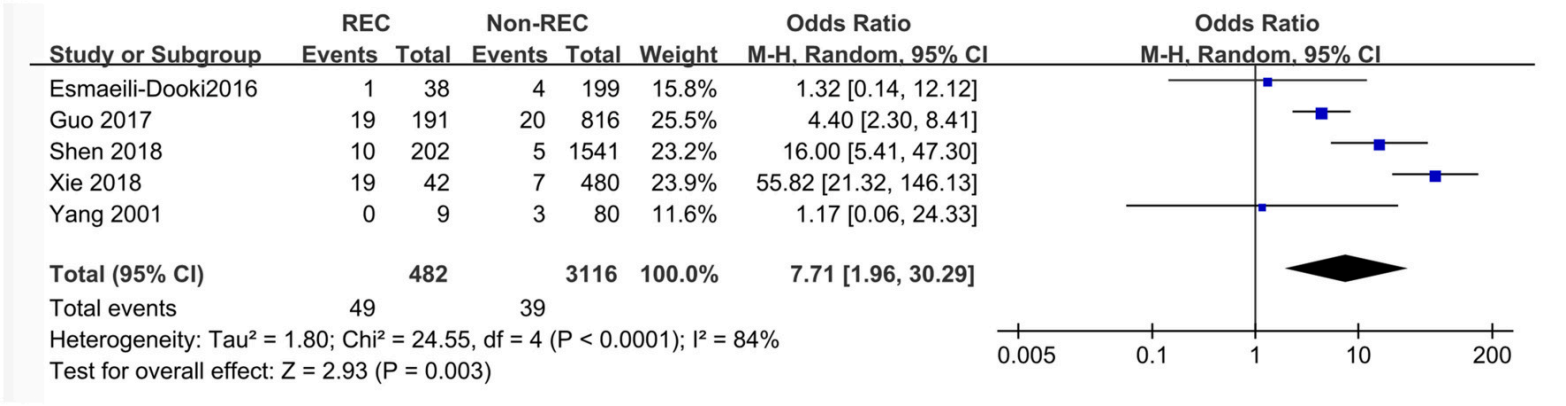
Forest plot showing pooled analyses of PLP and RI.

Data regarding the presence of PLP in participants were extracted from four studies. Among these, Guo et al. (6) and Xie et al. (12) reported a higher prevalence of PLP in RI patients, while the other two did not. This meta-analysis demonstrated that PLP may influence the presence of RI in a way, but it was not statistically significant (OR = 7.71 [1.96,30.29], *P* = 0.003) (Figure 7).

### Location of the Mass

**Figure 8:**
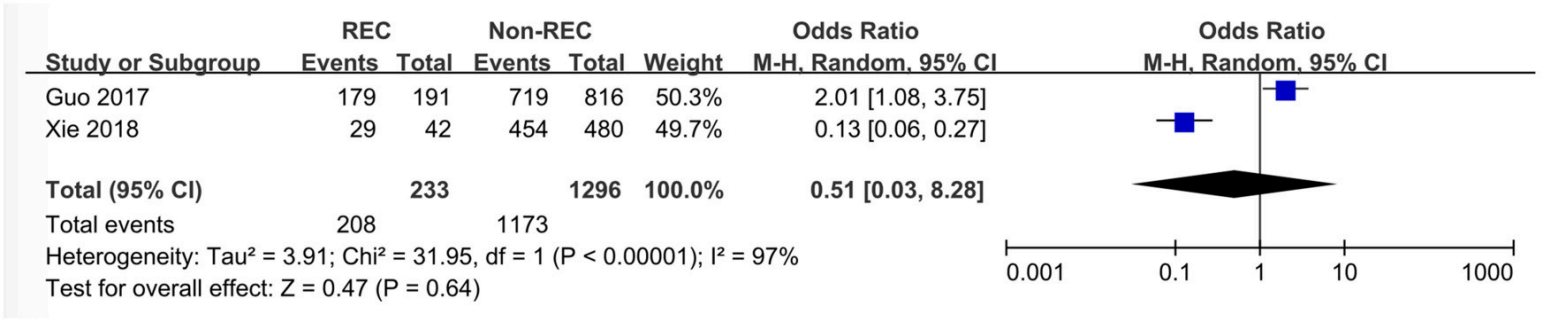
Forest plot showing pooled analyses of right abdominal mass and RI.

Data about location of the mass were extracted from only two studies and “right abdominal mass” was defined as “mass located in the right abdominal portion.” It is of interest that the conclusions in the two studies were contrary; Guo et al. (6) thought right abdominal mass was more likely to occur in RI patients, while Xie et al. (12) did not. This meta-analysis pooled their data and presented that there was no association between the presence of RI and location of the mass (OR = 0.51 [0.03, 8.28], *P* = 0.64) (Figure 8).

### Age

Because of the heterogeneity of the data, age in each of the studies could not be statistically pooled; thus, qualitative evaluation based on six related studies was taken. Both Guo et al. (6) and Wang et al. (5) reported that RI was more likely to occur in children aged above 1 year. Similarly, Kim et al. (7) and Xie et al. (12) thought children aged above 2 years were more susceptible to develop RI. Vo et al. (8) observed that there was no significant association between the presence of RI and atypical age, which was defined as either <6 months or >36 months.

## Discussion

### Summary of Main Findings

This meta-analysis included ten studies in which 1,273 children who recovered from intussusception with a recurrence and 10,685 without recurrence were enrolled. The following potential risk factors of RI were investigated: sex, fever, blood in stool, abdominal pain, vomiting, pathological lead point (PLP), and location of the mass. The predictive risk factors of RI identified were age and the prevalence of vomiting.

Of the five studies reviewed to investigate the influence of age in RI, two studies (5, 6) reported that RI was more likely to occur in children aged above 1 year, and the other two studies (7, 12) concluded that children aged above 2 years were more susceptible to develop RI. However, one study (8) observed that there was no significant association between the presence of RI and atypical age (<6 months or >36 months), but this finding is not contradictory to that in previous studies. Children aged <6 months showed a lower prevalence of RI, while those aged above 36 months showed a definitely higher prevalence, according to the findings in the other four studies. Thus, the average prevalence of RI in the atypical age groups would likely be neutral and children who were relatively older (over 1 or 2 years of age) may have a higher risk of recurrence.

The prevalence of vomiting was found to be lower in RI patients compared with patients in the control group. The other potential risk factors, including sex, the presence of blood in stool, fever, abdominal pain, right abdominal mass, and PLP failed to show significant association with RI in this meta-analysis. However, PLP may still be one of the potential risk factors of RI as it shows a result very close to statistical point (*P* = 0.06).

In addition to what has been presented here, this study also provided some other information that could not be systematically reviewed. Imaging was a widely-used method in the diagnosis of intussusception. However, whether there is a role of imaging in distinguishing RI children from those without recurrence was unclear. Xie et al. (12) reported that the poor prognosis sign shown by ultrasonography could distinguish between the RI participants and the controls (*P* = 0.048), while Kim et al. (7) did not think that ultrasonography had any predictive function after assessing all the measurements such as thickness of bowel ≥10 mm (*P* = 0.07), number of entrapped lymph nodes ≥2 (*P* = 0.71), and size of entrapped lymph nodes ≥1 mm (P = 1.0). Besides, radiological risk factors were also proved to be irrelevant with RI in the study performed by Vo et al. (8).

Although Reijnen et al. (24) had reported that duration of symptoms >48 h was a significant predictor of failure of hydrostatic reduction, few studies stated that the duration of symptoms was associated with the recurrence of intussusception in children. In this meta-analysis, conflicting conclusions were drawn in terms of how duration of symptoms influenced the presence of RI: Guo et al. (6) stated that a higher proportion of children with RI had symptoms for <12 h, whereas Xie et al. (12) showed that more recurrences occurred with >48 h duration of symptoms. Therefore, large-scale studies are needed in the future to address this problem.

### Limitation

This study has some limitations: (1) While the majority of the included studies are from Asia, ethnic confounding factors still exist because of the other American studies. (2) Certain factors such as body temperature were recorded in different formats, which hinders statistical analysis. (3) The cause of RI in some patients is still unknown. Although 12,008 participants were enrolled in the study, the number of overlapped risk factors or predictive symptoms in each of the studies were too less to be analyzed statistically in a comprehensive way. However, it is the first meta-analysis to investigate the risk factors of RI after successful enema reduction in children, to the best of our knowledge, which will certainly have some practical clinical implications.

## Conclusion

The main conclusion of this meta-analysis was that children with the presence of fever and PLP may have a higher risk of recurrence following enema reduction for intussusception and the prevalence of vomiting was found to be lower in RI patients than in the control group. Therefore, older age, absence of vomiting, the presence of fever and PLP may not be good factors, at least in terms of the probability for the occurrence of RI, a fact which deserves the attention of clinicians.

## Author Contributions

XY, RT, SC, ZL, and JZ contributed to the study conception and design. XY, RT, SC, and ZL performed data acquisition. XY, RT, SC, ZL, and JZ performed analysis and data interpretation. XY and RT drafted the manuscript. JZ performed critical revision.

### Conflict of Interest Statement

The authors declare that the research was conducted in the absence of any commercial or financial relationships that could be construed as a potential conflict of interest.
